# Spiro-Twisted Benzoxazine Derivatives Bearing Nitrile Group for All-Solid-State Polymer Electrolytes in Lithium Batteries

**DOI:** 10.3390/polym14142869

**Published:** 2022-07-14

**Authors:** Jen-Yu Lee, Tsung-Yu Yu, Shih-Chieh Yeh, Nae-Lih Wu, Ru-Jong Jeng

**Affiliations:** 1 Institute of Polymer Science and Engineering, National Taiwan University, Taipei 106, Taiwan; so850306w@gmail.com (J.-Y.L.); d07549003@ntu.edu.tw (T.-Y.Y.); 2 Advanced Research Center for Green Materials Science and Technology, National Taiwan University, Taipei 106, Taiwan; 3 Department of Chemical Engineering, National Taiwan University, Taipei 106, Taiwan

**Keywords:** spiro-twisted, benzoxazine, semi-interpenetrating polymer network, lithium-ion batteries (LIBs)

## Abstract

In this study, two nitrile-functionalized spiro-twisted benzoxazine monomers, namely 2,2′-((6,6,6′,6′-tetramethyl-6,6′,7,7′-tetrahydro-2H,2′H-8,8′-spirobi[indeno[5,6-e][1,3]oxazin]-3,3′(4H,4′H)-diyl)bis(4,1-phenylene))diacetonitrile (TSBZBC) and 4,4′-(6,6,6′,6′-tetramethyl-6,6′,7,7′-tetrahydro-2H,2′H-8,8′-spirobi[indeno[5,6-e][1,3]oxazin]-3,3′(4H,4′H)-diyl)dibenzonitrile (TSBZBN) were successfully developed as cross-linkable precursors. In addition, the incorporation of the nitrile group by covalent bonding onto the crosslinked spiro-twisted molecular chains improve the miscibility of SPE membranes with lithium salts while maintaining good mechanical properties. Owing to the presence of a high fractional free volume of spiro-twisted matrix, the –CN groups would have more space for rotation and vibration to assist lithium migration, especially for the benzyl cyanide-containing SPE. When combined with poly (ethylene oxide) (PEO) electrolytes, a new type of CN-containing semi-interpenetrating polymer networks for solid polymer electrolytes (SPEs) were prepared. The PEO-TSBZBC and PEO-TSBZBN composite SPEs (with 20 wt% crosslinked structure in the polymer) are denoted as the BC20 and BN20, respectively. The BC20 sample exhibited an ionic conductivity (σ) of 3.23 × 10^−4^ S cm^−1^ at 80 °C and a Li^+^ ion transference number of 0.187. The LiFePO_4_ (LFP)|BC20|Li sample exhibited a satisfactory charge–discharge capacity of 163.6 mAh g^−1^ at 0.1 C (with approximately 100% coulombic efficiency). Furthermore, the Li|BC20|Li cell was more stable during the Li plating/stripping process than the Li|BN20|Li and Li|PEO|Li samples. The Li|BC20|Li symmetric cell could be cycled continuously for more than 2700 h without short-circuiting. In addition, the specific capacity of the LFP|BC20|Li cell retained 87% of the original value after 50 cycles.

## 1. Introduction

Lithium-ion batteries (LIBs) are widely used as energy storage devices in portable electronics devices because of their high energy density and long lifetime [1]. Currently, LIBs are considered the best option for energy storage in diverse applications, such as electric vehicles and portable electronic devices. However, traditional LIBs contain organic liquid electrolytes. Therefore, they have several drawbacks, including safety concerns related to short-circuiting, overcharging, thermal issues, and potential leakage of flammable organic solvents [2]. Therefore, an urgent need exists for developing more reliable and safer electrolyte systems. Solid polymer electrolytes (SPEs) are considered promising materials for developing safe LIBs [3,4,5,6]. SPEs typically contain moveable polymer chains with polar atoms, such as O, S, P, and N, at an ideal distance from the coordination site that can efficiently dissociate lithium salts [7,8]. Wright and coworkers [9,10] found that the polyethylene oxide (PEO) polymer matrix can be used as an ionic conductor after doping it with lithium salts; thus, PEO has become an irreplaceable material in SPE for decades, mainly because of its ethylene oxide (EO) segment. The ether oxygen atoms in the EO segment can interact with cations such as lithium ions, which makes PEO extremely dissociative for a variety of salts [11,12]. Moreover, the excellent chain flexibility and segmental mobility of the EO segment enable lithium-ion conduction [13,14]. SPEs have attracted considerable attention from researchers as a promising candidate for use in rechargeable batteries. Unfortunately, the high crystallinity of PEO considerably limits the conduction and migration of lithium ions, and its low mechanical strength at high temperature limits its practical applications. In addition, the possibility of dendrite-induced short-circuiting limits the potential of PEO for use in LIBs [15,16,17,18]. Several attempts have been made to obtain amorphous polyether matrices with adequate mechanical properties to facilitate their use in practical applications. For instance, researchers have used materials such as plasticizers [19,20], organic fillers [21,22,23], comb polymers [24,25], and interpenetrating polymer networks (IPNs) [26,27,28,29] as well as methods such as copolymerization [30,31] and cross-linking [32,33,34] to reduce the glass transition temperature and crystallinity of polymers. Cross-linked polymer networks effectively improve the mechanical properties and stability of SPEs. For example, IPN structures can be created in PEO through ultraviolet light exposure or thermal treatment. These structures conduct lithium ions and strengthen the polymer matrix, thereby improving the ionic conductivity and mechanical properties of SPEs.

In our previous work [29], the design of the semi-interpenetrating polymer network (s-IPN) structure in the PEO matrix by using the hexanol group containing spiro-twisted cross-linkable benzoxazine cross-linking agent TSBZ6D have preliminarily enhanced the mechanical property and the electrochemical stabilities. These SPEs were called PTx and the x was represented to the weight percentage of the TSBZ6D in the polymer matrix. The lithium-ion transportation will be improved thanks to the increasing fractional free volume in the entire polymer with appropriate size distribution by the spiro-twisted structure. Moreover, the polybenzoxazine s-IPN structure in the PEO matrix led to the impressive enhancement in the mechanical properties. The PT20 sample exhibited a maximum stress about 300% higher and an elongation about 3000% which was 11 times more than that of the PEO/LiTFSI electrolyte. The presence of a mechanically toughened network help to mitigate the propagation of lithium dendrite in LIBs during operation. However, satisfactory results in terms of electrochemical properties and the battery performances still can be further enhanced. For example, the Li|PT20|Li symmetric cell only could be continuously cycled for 200h (0.1 mA cm^−2^) in the lithium platting and stripping test along with the tLi^+^ of 0.17. Apart from that, the LFP|PT20|Li battery exhibited 80% retention of discharge specific capacity of its original value. In order to tune the electrochemical properties of SPEs, incorporating functional groups through the placement of polar subunits, such as acrylamide, acrylonitrile, maleic anhydride, and carbonate into the polymer chains was regarded as a useful way to increase ionic dissociation [35]. These polar subunits (like succinonitrile; SN) not only coordinate ions and thus promote the dissociation of lithium salts in SPEs but also effectively reduce crystallinity. The −C≡N bond is approximately 1.16 Å, which reflects the sp hybridization of the triply bonded carbon [36,37]. The polar nitrile group has a high dipole moment, dielectric constant, and strong electron-withdrawing ability, which indicates that it possesses high electrochemical stability over a wide electrochemical window and is suitable for battery applications [36]. Moreover, because the attractive force between cations and anions is inversely proportional to dielectric permittivity, the presence of nitrile groups with high dielectric permittivity possibly enhances the dissociation strength of lithium salts [7,38,39]. Many SPEs blended with SN [40,41,42] are frequently used as key ingredients in the electrolytes of LIBs. However, the physical blending of SN in polymer matrix always faces the loss problem during fabrication process, and the decline of mechanical problem due to plasticizer effect [43].

**Scheme 1:**
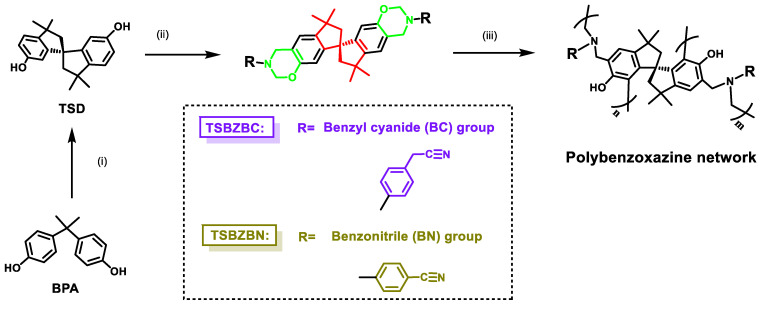
Synthetic route and ring-opening reaction of spiro-twist TSBZBN and TSBZBC: (i) methanesulfonic acid (135 °C, 4 h), (ii) 4-aminobenzonitrile or 4-aminobenzyl cyanide with paraformaldehyde in xylene reflux for 24 h, and (iii) thermal treatment (200 °C, 1.5 h).

Herein, we developed new cross-linkable benzoxazine molecules comprising a spiro-twisted core and benzyl cyanide or benzonitrile groups to prepare s-IPN electrolytes for LIBs. The synthetic route and ring-opening reaction of the nitrile group containing spirobisindane benzoxazine monomers 2,2′-((6,6,6′,6′-tetramethyl-6,6′,7,7′-tetrahydro-2H,2′H-8,8′-spirobi[indeno [5,6-e][1,3]oxazin]-3,3′(4H,4′H)-diyl)bis(4,1-phenylene))diacetonitrile (named as TSBZBC) and 4,4′-(6,6,6′,6′-tetramethyl-6,6′,7,7′-tetrahydro-2H,2′H-8,8′-spirobi[indeno [5,6-e][1,3]oxazin]-3,3′(4H,4′H)-diyl)dibenzonitrile (named as TSBZBN) are depicted in Scheme 1. The spiro-twisted core and cross-linking network derived from the cured benzoxazines play a crucial role in creating a high free volume and inhibiting the crystallinity of PEO matrices. In addition, the different functional groups of the polybenzoxazine network influence the migration of lithium ions. The TSBZBC and TSBZBN benzoxazine cross-linkers were incorporated into the PEO matrix to form an s-IPN structure for SPEs, and the performance of the resulting LIBs was evaluated.

## 2. Experimental Section

### 2.1. Materials

Bisphenol A (BPA) and methanesulfonic acid were purchased from Alfa Aesar (Ward Hill, MA, USA). Chloroform, anhydrous magnesium sulfate (MgSO_4_), 4-aminobenzonitrile, 4-aminobenzyl cyanide, and 1 N sodium hydroxide were purchased from TCI (Portland, OR, USA). Paraformaldehyde (extra pure grade) was purchased from Acros (Morris Plains, NJ, USA). The solid electrolyte polymer matrix, poly(ethylene oxide) (PEO) with Mw = 600,000–1,000,000 g mole^−1^ was supplied by Acros (Morris Plains, NJ, USA). Bis(trifluoromethane)sulfonimide lithium salt (LiTFSI), acetonitrile, xylene, and chloroform-d (CDCl_3_) were supplied by Sigma-Aldrich (Darmstadt, Germany). The cathode (lithium-ion phosphate, LiFePO_4_(LFP)) with a loading of 1.74 mAh cm^−2^, anode (lithium foil), and CR2032 coin cell were purchased from UBIQ Technology Co. (Taoyuan, Taiwan).

### 2.2. Synthesis of Spirobisindane-Containing TSBZBC and TSBZBN

TSBZBC and TSBZBN (Scheme 1) were synthesized in a manner similar to that described in our previous work [29]. BPA conversion in methanesulfonic acid (135 °C, 4 h) was performed to obtain the spiro-twist compound 3,3,3′,3′-tetramethyl-1,1′-spirobisindane-6,6′-diol (TSD). A mixture of 4-aminobenzyl cyanide (197.1 mmol, 26.0 g), paraformaldehyde (388.2 mmol, 11.8 g), xylene (300 mL), and TSD (97.1 mmol, 22.5 g) was prepared. This mixture was refluxed for 24 h to complete the reaction, and a yellow powder was then obtained through precipitation. The resulting product was dissolved in chloroform, washed thrice with 1 N NaOH, and then dried using anhydrous MgSO_4_. Subsequently, the chloroform was eliminated using a rotary evaporator to obtain yellow TSBZBC powder (yield: 70%). TSBZBN was prepared in the same manner as mentioned in the aforementioned text by replacing 4-aminobenzyl cyanide with 4-aminobenzonitrile.

TSBZBC:

^1^H nuclear magnetic resonance (NMR) (400 MHz, CDCl_3_): δ 7.23 (d, 2H), 7.11 (d, 2H), 6.79 (s, H), 6.25 (s, H), 5.29 (d, 2H), 4.67 (d, 2H), 3.68 (s, 2H), 2.24 (dd, 2H), 1.32 (d, 6H).

^13^C NMR (125 MHz, CDCl_3_): δ 153.6, 150.6, 148.3, 145.0, 128.8, 122.0, 119.6, 119.3, 118.3, 118.1, 112.4, 78.7, 59.5, 57.3, 50.7, 42.9, 31.8, 30.5, 22.8

Electrospray ionization mass spectrometry (ESI-MS): m/z value of C_41_H_40_N_2_O_2_Na [M + Na]^+^: calculated value = 643.8 and measured value = 643.2. The analytical data correspond to the theoretical data.

TSBZBN:

^1^H NMR (400 MHz, CDCl_3_): δ 7.58 (d, 2H), 7.08 (d, 2H), 6.83 (s, H), 6.28 (s, H), 5.32 (d, 2H), 4.73 (s, 2H), 2.27 (dd, 2H), 1.33 (d, 6H).

^13^C NMR (125 MHz, CDCl_3_): δ 153.5, 151.3, 150.8, 145.5, 133.6, 119.6, 119.0, 116.2, 114.4, 112.6, 102.5, 59.4, 57.3, 49.8, 43.0, 31.8, 30.4.

ESI-MS: m/z value of C_39_H_36_N_4_O_2_Na [M + Na]^+^: calculated value = 615.73 and measured value = 615.27. The analytical data correspond to the theoretical data.

### 2.3. Preparation of SPE Membranes

The SPEs were prepared following the procedure described in our previous work [29]. TSBZBC or TSBZBN was added to the 5% PEO solution (in acetonitrile) in a weight ratio of 20/80 ([TSBZBC or TSBZBN]/PEO). The SPEs prepared using TSBZBC and TSBZBN are hereafter denoted as BC20 and BN20, respectively. A certain quantity of LiTFSI (33 wt% of lithium salt in polymer electrolyte) was added to each blend. The BC20 and BN20 samples were poured onto a polytetrafluoroethylene plate and dried at 60 °C for 5 h to remove the solvent and obtain yellow-colored free-standing films. The color of these films changed to dark brown after thermal treatment at 200 °C for 1.5 h. An s-IPN structure comprising cross-linked polybenzoxazine/PEO was formed after the thermal treatment. The thicknesses of the SPE were 200–250 μm.

### 2.4. General Method

NMR spectra of the prepared compounds were recorded using a Bruker Avance III HD-600 MHz NMR spectrometer with CDCl_3_ as the d-solvent. The mass spectrum of the monomer was obtained through ESI-MS (AB SCIEX, QSTAR^®^ XL). The glass transition temperature (T_g_) of the SPEs and the thermal properties of the monomer were determined using a differential scanning calorimeter (TA Instruments, Q20) in a N_2_ atmosphere under a heating rate of 10 °C min^−1^. The dynamic mechanical analyzer (TA Instruments, Discovery DMA 850) was used to measure the mechanical properties of the SPE under a strain rate of 1% min^−1^. Electrochemical impedance spectroscopy (EIS) was performed for the SPEs over a frequency range of 1 Hz to 1 MHz (20–80 °C). Linear sweep voltammetry (LSV) scans of stainless steel (SS)|SPE|Li cells were performed at 0.2 mV s^−1^ to analyze the electrochemical stability of the prepared SPE samples (80 °C). The Li^+^ ion transference number (tLi^+^) of the SPE samples was determined according to the polarization of Li|SPE|Li cells under an applied voltage of 10 mV (80 °C). The EIS, LSV, and transference number tests were performed using a VSP potentiostat (Bio-Logic Science Instruments, SP-50, Seyssinet-Pariset, France). Lithium plating and stripping tests of the Li|SPE|Li symmetric cells and charge–discharge performance tests of the LFP|SPE|Li batteries were performed between 2.5 and 4.0 V by using a battery tester (CT-4008-5V6A Neware, Hongkong, China) (80 °C).

### 2.5. Electrochemical Characterization

The ionic conductivity of the SPE samples was determined using electrochemical impedance spectroscopy (EIS) at temperatures of 20–80 °C. The SPE samples (ranging in thickness from 150 to 170 μm) were cut into disks (area = 1.32 cm^2^). The resistance of the polymer electrolyte membrane (R_b_) was measured using the alternating current impedance method, where the amplitude of the applied voltage was 10 mV (1 MHz to 1 Hz). Thereafter, its ionic conductivity (σ) was evaluated using the equation $\sigma =d/(R_{b}\times A)$ [44].

σ: ionic conductivity (S cm^−1^)

d: thickness of membrane (cm)

A: area of SPE membrane (cm^2^)

R_b_: resistance (Ω)

The lithium-ion transference number (tLi^+^) was computed using the Bruce–Vincent–Evans equation as follows: ${\mathrm{tLi}}^{+}=\frac{I_{s}\left(\Delta U\Delta R_{0}I_{0}\right)}{I_{0}\left(\Delta U-R_{S}I_{S}\right)}$ [45,46]. Li|SPE|Li samples were tested at 80 °C by using a polarization voltage (ΔU) of 10 mV. The initial current (I_0_) and initial interfacial resistance (R_0_) before polarization were measured through EIS under the 10 mV (1 MHz to 1 Hz) amplitude of the applied voltage.

The electrochemical stability of the electrolyte was studied by conducting liner sweep voltammetry (LSV) measurements for the Li|SPE|stainless steel (SS) cells at 80 °C under a constant scan rate of 0.2 mV s^−1^ from 3 to 6 V. The stripping/plating tests of the Li|SPE|Li symmetric cells were performed at a current density of 0.1 mA cm^−2^ at 80 °C. The LFP|SPE|Li cell was assembled for the battery test, and galvanostatic charge–discharge cycling was performed over a voltage range of 2.5–4.0 V at current rates of 0.1–0.5 C. All the electrochemical measurements were performed using samples assembled in CR2032 coin cells in an Ar atmosphere to avoid the side effects of humidity and air, and the cells were maintained at 80 °C overnight before the tests.

## 3. Results and Discussion

### 3.1. Characterization of TSBZBC and TSBZBN

The nitrile-group-functionalized spiro-twist benzoxazine monomers TSBZBC and TSBZBN were prepared through a reaction among diol (TSD), primary amine containing a nitrile group (4-aminobenzonitrile, 4-aminobenzyl cyanide), and paraformaldehyde (Scheme 1). The synthesis route was similar to that described in our previous study [29]. The ^1^H NMR spectra of TSBZBC and TSBZBN are depicted in Figures S1 and S2, respectively. The chemical shifts at 1.27–1.38 and 2.18–2.32 ppm correspond to the –CH_3_ and –CH_2_– bonds of the spirobisindane structure. For TSBZBC, the chemical shifts of the cross-linkable benzoxazine group were confirmed by specific –CH_2_– signals of the oxazine ring, namely Ar-CH_2_-N and O-CH_2_-N, which appeared at 4.68 and 5.31 ppm [47], respectively, at positions f and e, respectively (Figure S1). For TSBZBN, the oxazine ring appeared at 4.73 and 5.33 ppm at positions f and e, respectively (Figure S2). Furthermore, according to the chemical shifts of the aromatic protons resonated at 7.12 (position d) and 7.25 (position b) ppm (TSBZBC) and at 7.1 (position d) and 7.55 (position b) ppm (TSBZBN), the apparent shift of position b in TSBZBN was mainly caused by the strong polar nitrile group on the benzene ring. Moreover, the aryl protons (-CH_2_-) at position g resonated at 3.68 ppm for TSBZBC. The results of ^13^C NMR characterization **(Figures S3 and S4**) further confirmed the structures of TSBZBC and TSBZBN. Characteristic peaks of the spirobisindane core were observed at 59.5, 57.3, 42.9, 31.8, and 30.5 ppm for TSBZBC and at 59.4, 57.3, 43.0, 31.7, and 30.5 ppm for TSBZBN. Moreover, characteristic peaks of oxazine rings were observed at 78.7 and 50.7 ppm for TSBZBC and at 102.5 and 49.9 ppm for TSBZBN [48]. The ESI-MS spectra of TSBZBC and TSBZBN (Figures S5 and S6, respectively) indicate their [M + Na]^+^ results. The aforementioned results confirm the successful synthesis of TSBZBC and TSBZBN.

### 3.2. Thermal Properties of TSBZBC, TSBZBN, and Their SPEs

**Figure 1:**
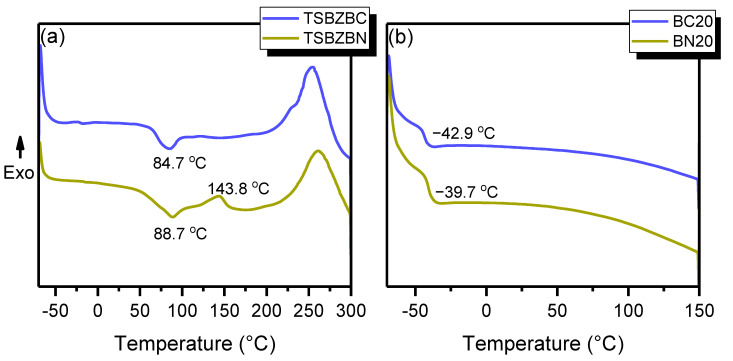
Differential scanning calorimetry thermograms of the (**a**) TSBZBC and TSBZBN monomers and (**b**) BC20 and BN20 electrolytes.

Figure 1 shows the differential scanning calorimetry thermogram used to investigate the thermal transitions of the synthesized s-IPN SPEs and their precursor in the temperature range of −70 to 150 °C at a scan rate of 10 °C min^−1^. The ring-opening cross-linking reaction of the benzoxazine segment occurs at about 200–250 °C [49,50,51,52,53,54]. According to the monomer reaction scan results presented in Figure 1a, an exothermal peak starting at 200 °C and reaching its maximum value at about 260 °C is present in the thermograms of the two aforementioned benzoxazine monomers. This phenomenon implies that the thermal cross-linking reaction started in the aforementioned temperature zone, which is consistent with the condition described in the literature. In addition, distinct crystallization temperature (T_c_) (143.8 °C) and melting point (T_m_) (88.7 °C) peaks were observed for the TSBZBN monomer. However, no T_c_ peak was observed for the TSBZBC monomer possibly because of the presence of an additional sigma bond between the benzene ring and the nitrile group of the benzyl cyanide group. The rotation of the single bond of the benzyl cyanide group could obstruct the packing and movement of the monomer. Moreover, because the TSBZBN monomer had a higher T_m_ value than did the TSBZBC monomer, its benzene ring was stacked more easily than that of the TSBZBC monomer.

Figure 1b shows the thermal properties of the two SPEs prepared using the TSBZBC and TSBZBN monomers as the cross-linking agents, respectively. No T_c_ or T_m_ peaks were observed for the BC20 and BN20 samples. Thus, the s-IPN structure effectively inhibited PEO crystallinity, which is consistent with the results of our previous study [29]. In the series of functionalized s-IPN SPEs, the PT20, BC20, and BN20 SPEs had the T_g_ in the same order. Among all, the BN20 exhibited the largest T_g_ of −39.7 °C owing to the incorporation of the aromatic ring and the higher possibility of packing ability. Nevertheless, a lower T_g_ value was observed for the BC20 sample than for the BN20 sample possibly because of the easier movement of the functional group in the BC20 sample. In addition, the mechanical properties of the SPE samples were measured by a dynamic mechanical analyzer (DMA). As shown in Figure S7, the BC40 sample exhibited 0.38 MPa in tensile stress and 40% in tensile strength. However, the BC20 sample possessed extreme elastomeric properties so that its mechanical properties measurement exceeded the DMA detection limit.

### 3.3. Lithium-Ion Conductivity

**Figure 2:**
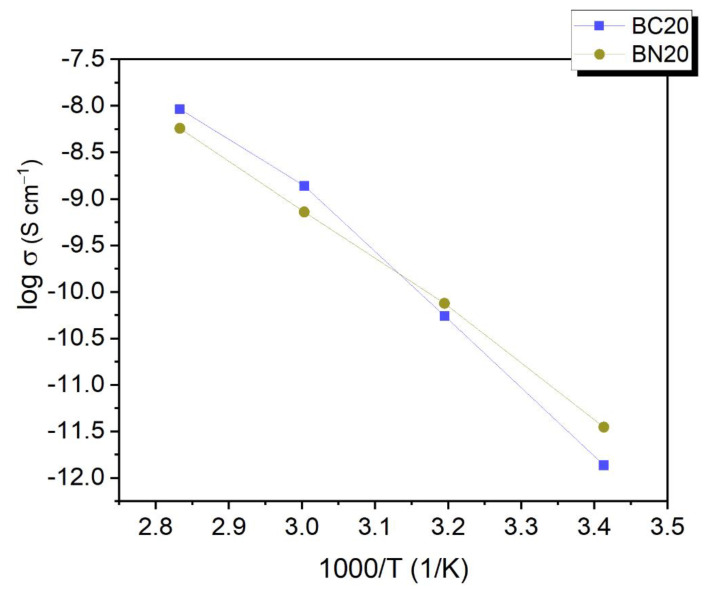
Ionic conductivities of the BC20 and BN20 samples at various temperatures.

The ionic conductivities of SPEs were decreased with increasing crosslinked degree of polybenzoxazine due to the lower mobility of the polymer segments (Table S1). The ionic conductivities, processibility, and mechanical properties were taken into account for choosing the SPE sample for further study. Based on the above, the BC20 and BN20 samples were chosen for electrochemical performance tests. SPEs should have good electrochemical performance to ensure that they can be used in practical applications. As depicted in Figure 2, the ionic conductivities of these newly developed s-IPN SPEs were measured in the temperature range of 20–80 °C. The ionic conductivities of the SPEs increased with increasing temperature because of the faster transfer of isolated ions and higher mobility of polymer chain segments at elevated temperatures [55,56,57]. The ionic conductivities of the samples had the same order at various temperatures, and their magnitudes were higher than 10^−4^ S cm^−1^ at 80 °C ($3.23\times 10^{-4}$ S cm^−1^ for the BC20 sample and $2.63\times 10^{-4}$ S cm^−1^ for the BN20 sample). Moreover, the relatively high ionic conductivities of the BC20 sample at temperatures exceeding 80 °C can possibly be ascribed to the presence of an additional sigma bond in the benzyl cyanide group compared to that in the benzonitrile group in the BN20 sample. The rotation of this single bond could obstruct packing and result in the easier movement of molecules. This result is consistent with the thermal properties presented in Figure 1.

A comparison of the effects of different functional groups on the ionic conductivities of the SPEs is summarized in Table 1. The incorporation of nitrile-group functionalized spiro-containing polybenzoxazine networks, along with the presence of abundant intermolecular interactions between the -OH group of polybenzoxazine, nitrile group and LiTFSI, will enhance the dissociation of the lithium-salt. However, in the samples with higher contents of aromatic rings (BC20 and BN20) compared with that hexanol side group series PT20 SPE, the migration of charge carriers might be hindered, which might lower their ionic conductivities marginally. Moreover, the difference in the molecular structure between the BC20 and BN20 sample at a smaller scale is responsible for the higher ionic conductivity of BC20 sample.

### 3.4. Lithium-Ion Transference Number (tLi^+^) and Electrochemical Stability

**Figure 3:**
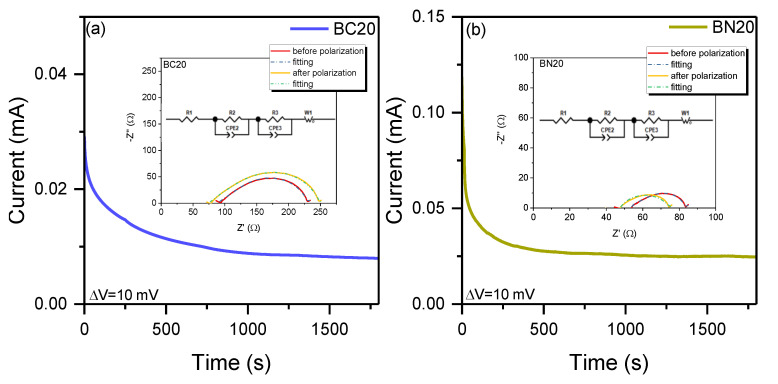
Time-dependent response of direct current polarization potential obtained for the (**a**) Li|BC20|Li and (**b**) Li|BN20|Li cells at 80 °C.

A higher Li^+^ transference number (tLi^+^) represents more restricted anion movement, which is a crucial factor affecting LIBs. A higher tLi^+^ can decrease the polarization effect during the charging–discharging process [7,58,59]. Figure 3 shows the change in current over 2000 s after polarization of the Li|SPE|Li cells at 10 mV with an EIS probe. The tLi^+^ values of the BC20 and BN20 samples were calculated to be 0.187 and 0.143, respectively, by using the Bruce–Vincent–Evans equation. The impedance data of the Li|SPE|Li cells were fitted to obtain the equivalent circuit by using the inserted model of the circuit depicted in Figure 3. R1 represents the bulk resistance of the electrolyte whereas the interface resistance such as the solid electrolyte interface formed on the lithium electrode (R2) and the charge transfer resistance (R3) lead to the diameter of the semicircle. The charge transport at low frequencies used a short Warburg element (W1) to describe.

Furthermore, the inclusion of different nitrile moieties, namely benzyl cyanide and benzonitrile, which have different electron-withdrawing abilities into the s-IPN structure, influences the polarization of lithium symmetric cells. A rapid decay in the current density of the BN20 electrolyte yielded a current ratio between the initial (I_0_) and steady (I_SS_) states of 0.208, which is consistent with the values obtained for most PEO-based electrolytes in the literature [60,61]. The current ratio of the BC20 electrolyte was 0.268. The decrease in cell polarization caused by the weaker electron-withdrawing ability of the BC20 electrolyte is especially noteworthy. The two aforementioned BC20 and BN20 samples are capable of blocking concentration polarization on the SPE surface [62,63].

**Figure 4:**
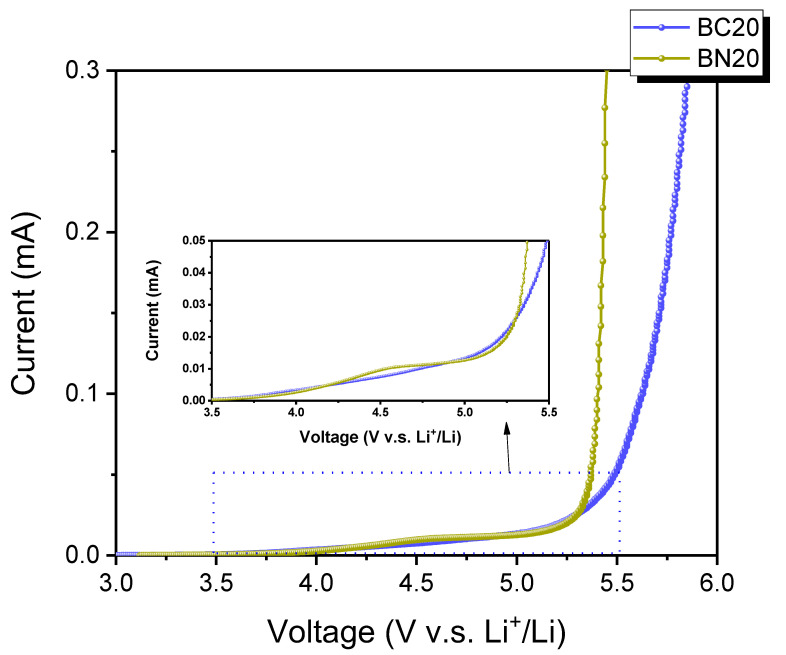
Linear sweep voltammetry curves of SS|BC20|Li and SS|BN20|Li samples at 80 °C.

The LSV curves recorded at potentials of 3–6 V (vs. Li^+^/Li) under a scanning rate of 0.2 mVs^−1^ at 80 °C (Figure 4) indicate the electrochemical stability windows of the BC20 and BN20 SPEs. The incorporation of the spiro-twist nitrile-side-functionalized polybenzoxazine s-IPN structure can enhance the oxidation resistance of the SPEs. No notable oxidation peak was detected at voltages of up to 5.38V in BC20 sample and in BN20 sample. Both the two samples exhibited a rather higher electrochemical window when compare with the PEO/LiTFSI electrolyte. According to the literature [29,64,65], oxidation of the PEO electrolyte starts at approximately 4 V. −C≡N···Li^+^···(EO)_n_ coordination can effectively delocalize the lone pair electrons in the EO units. The aforementioned results indicate that the s-IPN-bearing benzyl cyanide group is beneficial for stabilizing the PEG/Li^+^ coordination structure [61] because of its electron-withdrawing ability. Thus, the BC20 sample was oxidized at potentials higher than 5.38 V (vs. Li^+^/Li). This result revealed that the sample possessed anodic stability without considerable decomposition in the required charge–discharge potential range of 3–5 V, which indicated the suitability of this sample for further examination.

**Figure 5:**
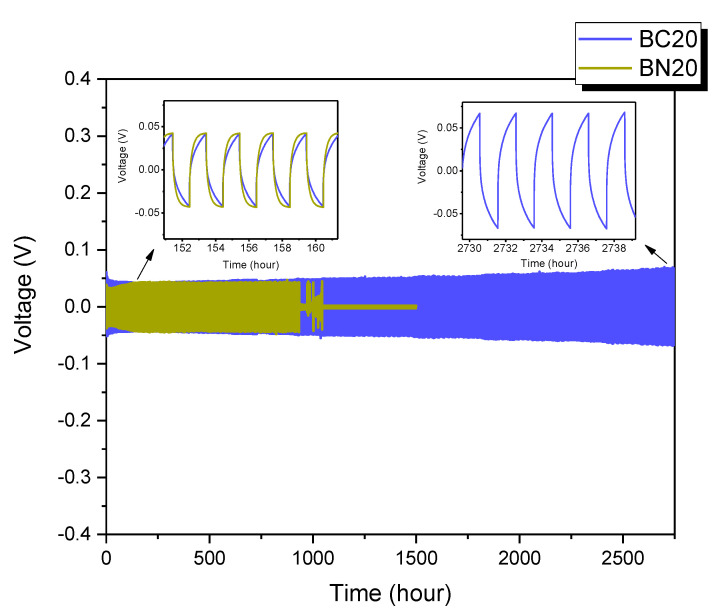
Lithium plating/stripping tests of Li|BC20|Li and Li|BN20|Li symmetric batteries at 80 °C.

To investigate whether SPE possessed the excellent stability required for its use in intimate contact with lithium metal anodes, we performed lithium plating/stripping tests on symmetric lithium cells under a constant current density of 0.1 mA cm^−2^ with a plating/stripping duration of 1 h at 80 °C (Figure 5). According to the galvanostatic discharge–charge profiles presented in Figure 5, the initial voltage polarization of the Li|BC20|Li and Li|BN20|Li cells occurred at around 60 and 38 mV, respectively. The Li|BC20|Li cell could be continuously cycled for more than 2700 h without short-circuiting, and a small voltage polarization was obtained, which indicated homogeneous contact and stable lithium plating/stripping behaviors between lithium metal and the BC20 sample. By contrast, the Li|BN20|Li cell could be continuously cycled for only approximately 900 h and the Li|PT20|Li cell for about 200 h [29]. This result confirms that the incorporation of the aforementioned nitrile-group-functionalized spiro-twisted polybenzoxazine s-IPN structures can solve the problems caused by uncontrollable lithium dendrite growth in a pristine PEO sample, as described in the literature. Moreover, the rotatable highly polar nitrile group might enhance the transportation of lithium ions; therefore, the BC20 electrolyte was more stable.

### 3.5. Electrochemical Performance

**Figure 6:**
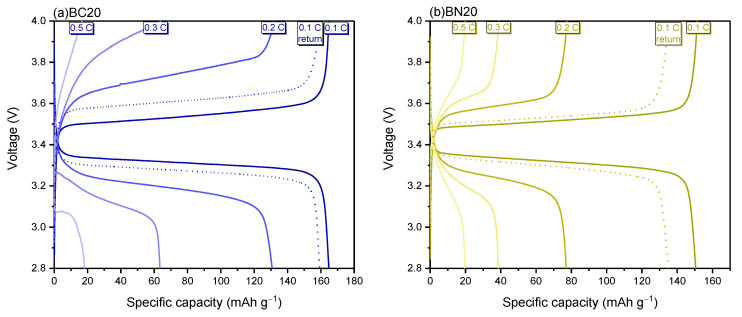
Charge–discharge profiles of the LFP|BC20|Li and LFP|BN20|Li cells at 80 °C.

**Figure 7:**
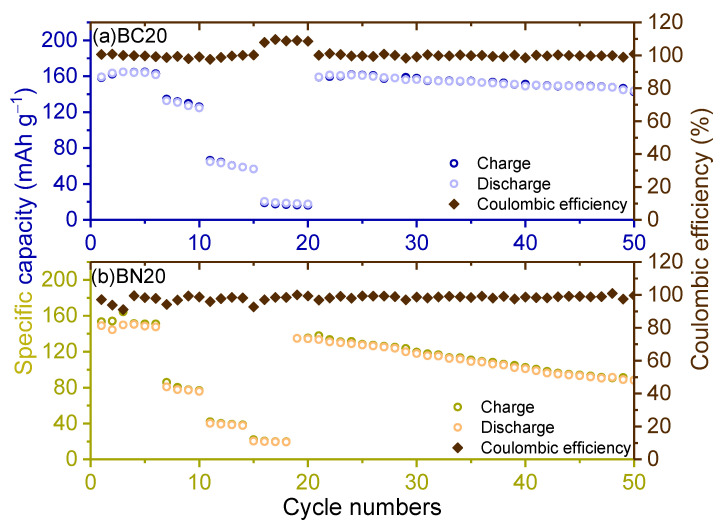
C-rate and cycling performance of the LFP|BC20|Li and LFP|BN20|Li cells at 80 °C.

The cell performances of the BC20 and BN20 samples were evaluated employing an LFP cathode and a lithium metal anode. The potential–capacity curves of the LFP|BC20|Li and LFP|BN20|Li cells are presented in Figure 6. The LFP|BC20|Li cell exhibited an initial discharge capacity of 163.6 mAh g^–1^ with a coulombic efficiency of approximately 99% (Figure 6a). Its specific capacity decreased as the current rate increased, and the specific capacity subsequently increased as the current rate decreased to 0.1 C (163.6, 134.5, 64.8, and 20.5 mAh g^−1^ at 0.1 C, 0.2 C, 0.3 C, and 0.5 C, respectively, and 159.1 mAh g^−1^ when returning backing to 0.1 C), which indicated the satisfactory rate capability of the cell. Moreover, the LFP|BN20|Li cell exhibited an initial discharge capacity of 149 mAh g^–1^ with a coulombic efficiency of approximately 97% (Figure 6b). The specific capacity of this cell decreased with increasing current rates, and it subsequently increased when current rate was decreased to 0.1 C (149, 80.8, 40.2, and 20.3 mAh g^–1^ at 0.1 C, 0.2 C, 0.3 C, and 0.5 C, respectively, and 134.9 mAh g^–1^ when returning back to 0.1 C). The potential–capacity curves at the current rates of 0.1–0.2, which were recorded during the charge–discharge processes of the BC20 and BN20 samples, exhibit a stable and clear voltage plateau arising from the Li/Li^+^ redox reaction. This observation indicated a good reversible process in the battery. Moreover, the consumption of lithium ions during the cycling process led to the polarization effect and a partly irreversible electrode reaction. Furthermore, the thickening of the solid electrolyte interphase film led to a decrease in the specific discharge capacity of the SPE at high C-rates [66]. This functional s-IPN structure design can help alleviate the overcharging phenomenon in pristine PEO electrolytes, which might be caused by the poor mechanical properties of the PEO electrolyte and results in the formation of lithium dendrites [67]. According to the ionic conductivities and tLi^+^ results presented in Figure 2 and Figure 3, respectively, lower ionic conductivities and tLi^+^ values of the BN20 sample resulted in higher internal resistance and lower reaction kinetics of active materials during the generation of lithium ions and electrons. Therefore, at the same current rate, the battery capacity of the LFP|BN20|Li cell was lower than that of the LFP|BC20|Li cell. Furthermore, the C-rate and cyclic performance of the assembled cells are presented in Figure 7. The cycle stability of the LFP|BC20|Li cell was superior to that of the LFP|BN20|Li cell. After 50 charge–discharge cycles, the LFP|BC20|Li cell retained 87.2% of its discharge capacity in the initial state, whereas the LFP|BN20|Li cell retained only 59.2% of its discharge capacity in the initial state. It is important to note that an abnormality of a coulombic efficiency larger than 100% at 0.5 C was found in the cycles of 16–20 for the LFP|BC20|Li cell. The rather thick BC20 films (200–250 μm) in this study might cause the concentration polarization phenomenon and lead to a coulombic efficiency larger than 100% at 0.5 C. Therefore, a BC20 sample of (150 μm) was further fabricated for the lithium battery with the same operating condition. The abnormality of a coulombic efficiency larger than 100% at 0.5 C was not observed for the battery with a thinner SPE film (Figures S8 and S9).

The electrochemical performance comparison of SPEs in this work and those reported in the literature is shown in Table S2 [29],[65],[68],[69],[70],[71],[72] . The SPEs in this work exhibited comparable electrochemical properties for the LIBs. Especially, the BC20 sample in the platting and stripping test presented a continuous cycle for over 2700 h (0.1 mA cm^−2^). The presence of benzyl cyanide groups in the BC20 sample would certainly bring about better compatibility among polybenzoxazine networks, PEO matrix, and lithium salts [61]. The incorporation of the rather flexible high polar groups such as benzyl cyanide groups, instead of benzonitrile groups, in the SPE sample might provide a viable way for fabricating a high-performance lithium battery. In addition, when compared with our previous work [29], the electrochemical properties were significantly improved by replacing the hexanol group-containing spiro-twisted benzoxazine (TSBZ6D) with the benzyl cyanide group-containing spiro-twisted benzoxazine (TSBZBC). The benzyl cyanide group-containing BC20 sample could be continuously cycled for over 2700 h in the plating/stripping test along with the tLi^+^ of 0.187, whereas the hexanol group-containing PT20 sample could be continuously cycled for only 200 h in the plating/stripping test along with the tLi^+^ of 0.170. In addition, the LFP|BC20|Li battery possessed 87.2% whereas the LFP|PT20|Li battery possessed 80.0% of its initial discharge capacity after 50 cycles.

## 4. Conclusions and Outlook

In this study, lithium batteries based on two SPEs, benzyl cyanide-containing BC20 and benzonitrile-containing BN20, were developed. The BC20 sample exhibited an ionic conductivity of 3.23 × 10^−4^ S cm^−1^ along with a tLi^+^ of 0.187, whereas the BN20 sample exhibited an ionic conductivity of 2.63 × 10^−4^ S cm^−1^ along with the tLi^+^ of 0.143. Apart from that, the BN20 sample can be continuously cycled for 900 h and the BC20 sample can be continuously cycled for over 2700 h in the plating/stripping test. The LFP|BC20|Li battery possessed 87.2% of its initial discharge capacity after 50 cycles. Better electrochemical properties were achieved by the benzyl cyanide-containing BC20 sample with relatively mobile nitrile groups. The robustness of the proposed LFP|BC20|Li cell is ascribed to interactions between abundant hydrogen bonding sites and polar species (such as TFSI^-^ anions), and −C≡N···Li^+^···(EO)_n_ coordination can effectively delocalize the lone pair electrons of EO units that immobilize anions, thereby enhancing the transfer of lithium ions. Thus, through the suitable functional structural design of the spiro-twisted s-IPN structures in SPEs, one can develop advanced polymer electrolytes with synergistic improvements in electrochemical stability, ionic conductivity, and battery performance.

## Figures and Tables

**Table 1:** Ionic conductivity (S cm^−1^) values for the BC20, BN20, and PT20 samples at various temperatures (°C).

		Sample	BC20	BN20	PT20 [29]
	Ionic Conductivities (S cm^−1^)				
Temperature (°C)					
RT			$\;7.047\times 10^{-6}$	$1.060\times 10^{-5}$	$1.749\times 10^{-5}$
40			$3.507\times 10^{-5}$	$4.011\times 10^{-5}$	$6.138\times 10^{-5}$
60			$1.419\times 10^{-4}$	$1.072\times 10^{-4}$	$1.769\times 10^{-4}$
80			$3.238\times 10^{-4}$	$2.633\times 10^{-4}$	$3.539\times 10^{-4}$

RT: room temperature

## Data Availability

Not applicable.

## Supplementary Materials

The following supporting information can be downloaded at: https://www.mdpi.com/article/10.3390/polym14142869/s1, Figure S1: ^1^H NMR spectrum of TSBZBC, Figure S2: ^1^H NMR spectrum of TSBZBN, Figure S3: The ^13^C NMR spectra for TSBZBC, Figure S4: The 13C NMR spectra for TSBZBN, Figure S5: The ESI mass spectra of TSBZBC, Figure S6: The ESI mass spectra of TSBZBN, Figure S7: DMA stress−strain curves of the BC20 and BC40 sample, Figure S8: Cycling performance of the LFP|BC20|Li cell at 0.5C (80 °C), Figure S9: Charge/discharge profile of the LFP|BC20|Li cell at 80 °C, Table S1: The ionic conductivities result of the BC10, BC20, BC30, and BC40 samples at various temperatures, Table S2: Comparison of SPEs in this work and those reported in literature.

## References

[1] Li M., Lu J., Chen Z., Amine K. (2018). 30 Years of Lithium-Ion Batteries. Adv. Mater..

[2] Bandhauer T.M., Garimella S., Fuller T.F. (2011). A Critical Review of Thermal Issues in Lithium-Ion Batteries. J. Electrochem. Soc..

[3] Ngai K.S., Ramesh S., Ramesh K., Juan J.C. (2016). A Review of Polymer Electrolytes: Fundamental, Approaches and Applications. Ionics.

[4] Agrawal R.C., Pandey G.P. (2008). Solid Polymer Electrolytes: Materials Designing and All-Solid-State Battery Applications: An Overview. J. Phys. D Appl. Phys..

[5] An Y., Han X., Liu Y., Azhar A., Na J., Nanjundan A.K., Wang S., Yu J., Yamauchi Y. (2022). Progress in Solid Polymer Electrolytes for Lithium-Ion Batteries and Beyond. Small.

[6] Sun J., Zou Y., Gao S., Shao L., Chen C. (2020). Robust Strategy of Quasi-Solid-State Electrolytes to Boost the Stability and Compatibility of Mg Ion Batteries. ACS Appl. Mater. Interfaces.

[7] Kim S., Le Mong A., Kim D. (2022). Accelerated Ion Conduction by Co-Grafting of Poly(ethylene glycol) and Nitrile-Terminated Ionic Liquid on Poly(arylene ether sulfone) for Solid Electrolyte Membranes for Lithium Ion Battery. J. Power Sources.

[8] Amaral F.A., Sousa R.M., Morais L.C.T., Rocha R.G., Campos I.O., Fagundes W.S., Fonseca C.N.P., Canobre S.C. (2015). Preparation and Characterization of the Porous Solid Polymer Electrolyte of PAN/PVA by Phase Inversion. J. Appl. Electrochem..

[9] Wright P.V. (1975). Electrical Conductivity in Ionic Complexes of Poly(ethylene oxide). Brit. Polym. J..

[10] Wright P.V. (1998). Polymer Electrolytes—The Early Days. Electrochim. Acta.

[11] Xue Z., He D., Xie X. (2015). Poly(ethylene oxide)-Based Electrolytes for Lithium-Ion Batteries. J. Mater. Chem. A.

[12] Jiang Y., Yan X., Ma Z., Mei P., Xiao W., You Q., Zhang Y. (2018). Development of the PEO Based Solid Polymer Electrolytes for All-Solid State Lithium Ion Batteries. Polymers.

[13] Meyer W.H. (1998). Polymer Electrolytes for Lithium-Ion Batteries. Adv. Mater..

[14] Yang M., Hou J. (2012). Membranes in Lithium Ion Batteries. Membranes.

[15] Magistris A., Singh K. (1992). PEO-Based Polymer Electrolytes. Polym. Int..

[16] Quartarone E., Mustarelli P., Magistris A. (1998). PEO-Based Composite Polymer Electrolytes. Solid State Ionics.

[17] Xu K. (2014). Electrolytes and Interphases in Li-ion Batteries and Beyond. Chem. Rev..

[18] Brissot C., Rosso M., Chazalviel J.-N., Baudry P., Lascaud S. (1998). In Situ Study of Dendritic Growth in Lithium/PEO-Salt/Lithium Cells. Electrochim. Acta.

[19] Bandara T.M.W.J., Gunasekara L.B.E., Gunathilake S.M.S., Mellander B.E. (2022). Transport Parameters of Charge Carriers in PEO-LiTf-Based, Plasticized, Composite, and Plasticized-Composite Electrolytes Intended for Li-Ion Batteries. Ionics.

[20] Sharma J.P., Singh V. (2020). Influence of High and Low Dielectric Constant Plasticizers on the Ion Tansport Properties of PEO: NH_4_HF_2_ Polymer Electrolytes. High Perform. Polym..

[21] Shi R., Liao K., Wang C. (2022). One-Dimensional Metal–Organic Framework-Reinforced Gel Polymer Electrolyte Enables a Stable Li Metal Battery. Asia-Pac. J. Chem. Eng..

[22] Barbosa J.C., Gonçalves R., Costa C.M., de Zea Bermudez V., Fidalgo-Marijuan A., Zhang Q., Lanceros-Méndez S. (2021). Metal–Organic Frameworks and Zeolite Materials as Active Fillers for Lithium-Ion Battery Solid Polymer Electrolytes. Mater. Adv..

[23] Ye F., Liao K., Ran R., Shao Z. (2020). Recent Advances in Filler Engineering of Polymer Electrolytes for Solid-State Li-Ion Batteries: A Review. Energy Fuels.

[24] Daigle J.-C., Vijh A., Hovington P., Gagnon C., Hamel-Pâquet J., Verreault S., Turcotte N., Clément D., Guerfi A., Zaghib K. (2015). Lithium Battery with Solid Polymer Electrolyte Based on Comb-Like Copolymers. J. Power Sources.

[25] Wei Z., Chen S., Wang J., Wang Z., Zhang Z., Yao X., Deng Y., Xu X. (2018). Superior Lithium Ion Conduction of Polymer Electrolyte with Comb-Like Structure via Solvent-Free Copolymerization for Bipolar All-Solid-State Lithium Battery. J. Mater. Chem. A.

[26] More S.S., Khupse N.D., Ambekar J.D., Kulkarni M.V., Kale B.B. (2022). Ionic Liquid-Supported Interpenetrating Polymer Network Flexible Solid Electrolytes for Lithium-Ion Batteries. Energy Fuels.

[27] Ha H.-J., Kil E.-H., Kwon Y.H., Kim J.Y., Lee C.K., Lee S.-Y. (2012). UV-Curable Semi-Interpenetrating Polymer Network-Integrated, Highly Bendable Plastic Crystal Composite Electrolytes for Shape-Conformable All-Solid-State Lithium Ion Batteries. Energy Environ. Sci..

[28] Lu Q., Yang J., Lu W., Wang J., Nuli Y. (2015). Advanced Semi-Interpenetrating Polymer Network Gel Electrolyte for Rechargeable Lithium Batteries. Electrochim. Acta.

[29] Lee J.-Y., Yu T.-Y., Chung P.-H., Lee W.-Y., Yeh S.-C., Wu N.-L., Jeng R.-J. (2021). Semi-Interpenetrating Polymer Network Electrolytes Based on a Spiro-Twisted Benzoxazine for All-Solid-State Lithium-Ion Batteries. ACS Appl. Energy Mater..

[30] Xiao Q., Wang X., Li W., Li Z., Zhang T., Zhang H. (2009). Macroporous Polymer Electrolytes Based on PVDF/PEO-b-PMMA Block Copolymer Blends for Rechargeable Lithium Ion Battery. J. Membr. Sci..

[31] Sadoway D.R. (2004). Block and Graft Copolymer Electrolytes for High-Performance, Solid-State, Lithium Batteries. J. Power Sources.

[32] Falco M., Simari C., Ferrara C., Nair J.R., Meligrana G., Bella F., Nicotera I., Mustarelli P., Winter M., Gerbaldi C. (2019). Understanding the Effect of UV-Induced Cross-Linking on the Physicochemical Properties of Highly Performing PEO/LiTFSI-Based Polymer Electrolytes. Langmuir.

[33] Ueno M., Imanishi N., Hanai K., Kobayashi T., Hirano A., Yamamoto O., Takeda Y. (2011). Electrochemical Properties of Cross-Linked Polymer Electrolyte by Electron Beam Irradiation and Application to Lithium Ion Batteries. J. Power Sources.

[34] Gong Z., Zheng S., Zhang J., Duan Y., Luo Z., Cai F., Yuan Z. (2022). Cross-Linked PVA/HNT Composite Separator Enables Stable Lithium-Organic Batteries under Elevated Temperature. ACS Appl. Mater. Interfaces.

[35] Hou W.-H., Chen C.-Y. (2004). Studies on Comb-Like Polymer Electrolyte with a Nitrile Group. Electrochim. Acta.

[36] Hu P., Chai J., Duan Y., Liu Z., Cui G., Chen L. (2016). Progress in Nitrile-Based Polymer Electrolytes for High Performance Lithium Batteries. J. Mater. Chem. A.

[37] Karakida K.-i., Fukuyama T., Kuchitsu K. (1974). Molecular Structures of Hydrogen Cyanide and Acetonitrile as Studied by Gas Electron Diffraction. Bull. Chem. Soc. Jpn..

[38] Sai R., Ueno K., Fujii K., Nakano Y., Shigaki N., Tsutsumi H. (2017). Role of Polar Side Chains in Li^+^ Coordination and Transport Properties of Polyoxetane-Based Polymer Electrolytes. Phys. Chem. Chem. Phys..

[39] Verdier N., Lepage D., Prébé A., Aymé-Perrot D., Dollé M., Rochefort D. (2018). Crosslinker Free Thermally Induced Crosslinking of Hydrogenated Nitrile Butadiene Rubber. J. Polym. Sci. A Polym. Chem..

[40] Chen R., Liu F., Chen Y., Ye Y., Huang Y., Wu F., Li L. (2016). An Investigation of Functionalized Electrolyte using Succinonitrile Additive for High Voltage Lithium-Ion Batteries. J. Power Sources.

[41] Echeverri M., Kim N., Kyu T. (2012). Ionic Conductivity in Relation to Ternary Phase Diagram of Poly(ethylene oxide), Succinonitrile, and Lithium Bis(trifluoromethane)sulfonimide Blends. Macromolecules.

[42] Fan L.-Z., Hu Y.-S., Bhattacharyya A.J., Maier J. (2007). Succinonitrile as a Versatile Additive for Polymer Electrolytes. Adv. Funct. Mater..

[43] Gupta R.K., Kim H.-M., Rhee H.-W. (2011). Poly(ethylene oxide): Succinonitrile—A Polymeric Matrix for Fast-Ion Conducting Redox-Couple Solid Electrolytes. J. Phys. D Appl. Phys..

[44] Qian X., Gu N., Cheng Z., Yang X., Wang E., Dong S. (2001). Methods to Study the Ionic Conductivity of Polymeric Electrolytes using A.C. Impedance Spectroscopy. J. Solid State Electrochem..

[45] Choi H., Kim H.W., Ki J.-K., Lim Y.J., Kim Y., Ahn J.-H. (2017). Nanocomposite Quasi-Solid-State Electrolyte for High-Safety Lithium Batteries. Nano Res..

[46] Xu H., He Y., Zhang Z., Shi J., Liu P., Tian Z., Luo K., Zhang X., Liang S., Liu Z. (2020). Slurry-Like Hybrid Electrolyte with High Lithium-Ion Transference Number for Dendrite-Free Lithium Metal Anode. J. Energy Chem..

[47] Feng Z., Zeng M., Tan D., Lu X., Shen Y., Xu Q., Meng D. (2022). Two Photosensitive Chalcone-Based Benzoxazine Monomers and their High-Performance Polymers from Renewable Sources. J. Mater. Sci..

[48] Sini N.K., Azechi M., Endo T. (2015). Synthesis and Properties of Spiro-Centered Benzoxazines. Macromolecules.

[49] Ribeiro F.W.M., Rodrigues-Oliveira A.F., Correra T.C. (2019). Benzoxazine Formation Mechanism Evaluation by Direct Observation of Reaction Intermediates. J. Phys. Chem. A.

[50] Kiskan B., Yagci Y., Ishida H. (2008). Synthesis, Characterization, and Properties of New Thermally Curable Polyetheresters Containing Benzoxazine Moieties in The Main Chain. J. Polym. Sci. Part A Polym. Chem..

[51] Chang S.L., Lin C.H. (2010). Facile, One-Pot Synthesis of Aromatic Diamine-Based Benzoxazines and Their Advantages Over Diamines as Epoxy Hardeners. J. Polym. Sci. Part A Polym. Chem..

[52] Zhang K., Han M., Liu Y., Froimowicz P. (2019). Design and Synthesis of Bio-Based High-Performance Trioxazine Benzoxazine Resin via Natural Renewable Resources. ACS Sustain. Chem. Eng..

[53] Arslan M., Kiskan B., Yagci Y. (2015). Benzoxazine-Based Thermosets with Autonomous Self-Healing Ability. Macromolecules.

[54] Zu L.-W., Gao B.-C., Pan Z.-C., Wang J., Dayo A.Q., Liu W.-B. (2019). Amino-Functionalized Lead Phthalocyanine-Modified Benzoxazine Resin: Curing Kinetics, Thermal, and Mechanical Properties. Polymers.

[55] Yao W., Zhang Q., Qi F., Zhang J., Liu K., Li J., Chen W., Du Y., Jin Y., Liang Y. (2019). Epoxy Containing Solid Polymer Electrolyte for Lithium Ion Battery. Electrochim. Acta.

[56] Lu Y., Das S.K., Moganty S.S., Archer L.A. (2012). Ionic Liquid-Nanoparticle Hybrid Electrolytes and their Application in Secondary Lithium-Metal Batteries. Adv. Mater..

[57] Zhang J., Zhang H., Zhang Y., Zhang J., He H., Zhang X., Shim J.-J., Zhang S. (2019). Unveiling of the Energy Storage Mechanisms of Multi -Modified (Nb_2_O_5_@C)/rGO Nanoarrays as Anode for High Voltage Supercapacitors with Formulated Ionic Liquid Electrolytes. Electrochim. Acta.

[58] Wu B., Wang S., Evans Iv W.J., Deng D.Z., Yang J., Xiao J. (2016). Interfacial Behaviours Between Lithium Ion Conductors and Electrode Materials in Various Battery Systems. J. Mater. Chem. A.

[59] Zhang C., Shen L., Shen J., Liu F., Chen G., Tao R., Ma S., Peng Y., Lu Y. (2019). Anion-Sorbent Composite Separators for High-Rate Lithium-Ion Batteries. Adv. Mater..

[60] Pożyczka K., Marzantowicz M., Dygas J.R., Krok F. (2017). Ionic conductivity and lithium transference number of poly(ethylene oxide): LiTFSI system. Electrochim. Acta.

[61] Kim J., Jeong K.-J., Kim K., Son C.Y., Park M.J. (2022). Enhanced Electrochemical Properties of Block Copolymer Electrolytes with Blended End-Functionalized Homopolymers. Macromolecules.

[62] Du Z., Su Y., Qu Y., Zhao L., Jia X., Mo Y., Yu F., Du J., Chen Y. (2019). A Mechanically Robust, Biodegradable and High Performance Cellulose Gel Membrane as Gel Polymer Electrolyte of Lithium-Ion Battery. Electrochim. Acta.

[63] Liu M., Wang Y., Li M., Li G., Li B., Zhang S., Ming H., Qiu J., Chen J., Zhao P. (2020). A New Composite Gel Polymer Electrolyte Based on Matrix of PEGDA with High Ionic Conductivity for Lithium-Ion Batteries. Electrochim. Acta.

[64] Liu K., Zhang R., Sun J., Wu M., Zhao T. (2019). Polyoxyethylene (PEO)|PEO–Perovskite|PEO Composite Electrolyte for All-Solid-State Lithium Metal Batteries. ACS Appl. Mater. Interfaces.

[65] Yu T.-Y., Yeh S.-C., Lee J.-Y., Wu N.-L., Jeng R.-J. (2021). Epoxy-Based Interlocking Membranes for All Solid-State Lithium Ion Batteries: The Effects of Amine Curing Agents on Electrochemical Properties. Polymers.

[66] Liu C., Neale Z.G., Cao G. (2016). Understanding Electrochemical Potentials of Cathode Materials in Rechargeable Batteries. Mater. Today.

[67] Wen J., Yu Y., Chen C. (2012). A Review on Lithium-Ion Batteries Safety Issues: Existing Problems and Possible Solutions. Mater. Express.

[68] Zhang H., Liu C., Zheng L., Xu F., Feng W., Li H., Huang X., Armand M., Nie J., Zhou Z. (2014). Lithium Bis(fluorosulfonyl)imide/poly(ethylene oxide) Polymer Electrolyte. Electrochim. Acta.

[69] Jo Y.H., Li S., Zuo C., Zhang Y., Gan H., Li S., Yu L., He D., Xie X., Xue Z. (2020). Self-Healing Solid Polymer Electrolyte Facilitated by a Dynamic Cross-Linked Polymer Matrix for Lithium-Ion Batteries. Macromolecules.

[70] Ben youcef H., Garcia-Calvo O., Lago N., Devaraj S., Armand M. (2016). Cross-Linked Solid Polymer Electrolyte for All-Solid-State Rechargeable Lithium Batteries. Electrochim. Acta.

[71] Yuan H., Luan J., Yang Z., Zhang J., Wu Y., Lu Z., Liu H. (2020). Single Lithium-Ion Conducting Solid Polymer Electrolyte with Superior Electrochemical Stability and Interfacial Compatibility for Solid-State Lithium Metal Batteries. ACS Appl. Mater. Interfaces.

[72] Huang S., Cui Z., Qiao L., Xu G., Zhang J., Tang K., Liu X., Wang Q., Zhou X., Zhang B. (2019). An in-situ polymerized solid polymer electrolyte enables excellent interfacial compatibility in lithium batteries. Electrochim. Acta.

