# Supplementary material for: Spiro-Twisted Benzoxazine Derivatives Bearing Nitrile Group for All-Solid-State Polymer Electrolytes in Lithium Batteries
Source: Polymers (Basel). 2022 Jul 14;14(14):2869. doi: 10.3390/polym14142869 (PMC9317537; doi:10.3390/polym14142869)
Supplement: Supplementary file 1 [file polymers-14-02869-s001.zip › polymers-1771302-supplementary.pdf]

Supporting information

Spiro-Twisted Benzoxazine Derivatives bearing Nitrile Group for All-Solid-State Polymer Electrolytes in Lithium Batteries

Jen-Yu Lee[a], Tsung-Yu Yu[a], Shih-Chieh Yeh[a,b], Nae-Lih Wu[b,c*], Ru-Jong Jeng[a,b*]*

---

[a]Institute of Polymer Science and Engineering, National Taiwan University, Taipei 106, Taiwan

[b]Advanced Research Center for Green Materials Science and Technology, National Taiwan University, Taipei 106, Taiwan

[c]Department of Chemical Engineering, National Taiwan University, Taipei 106, Taiwan

*Corresponding Author:

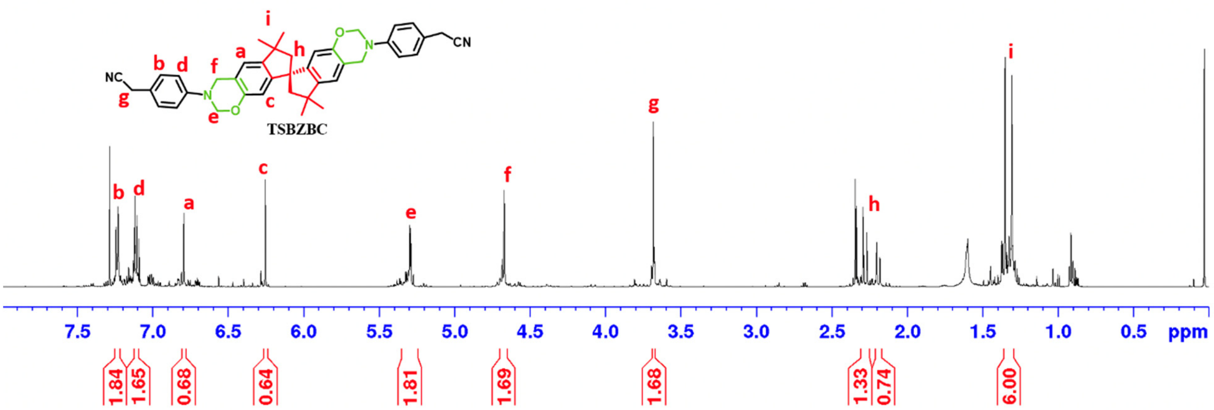

Figure S1. ^{1}H NMR spectrum of TSBZBC.

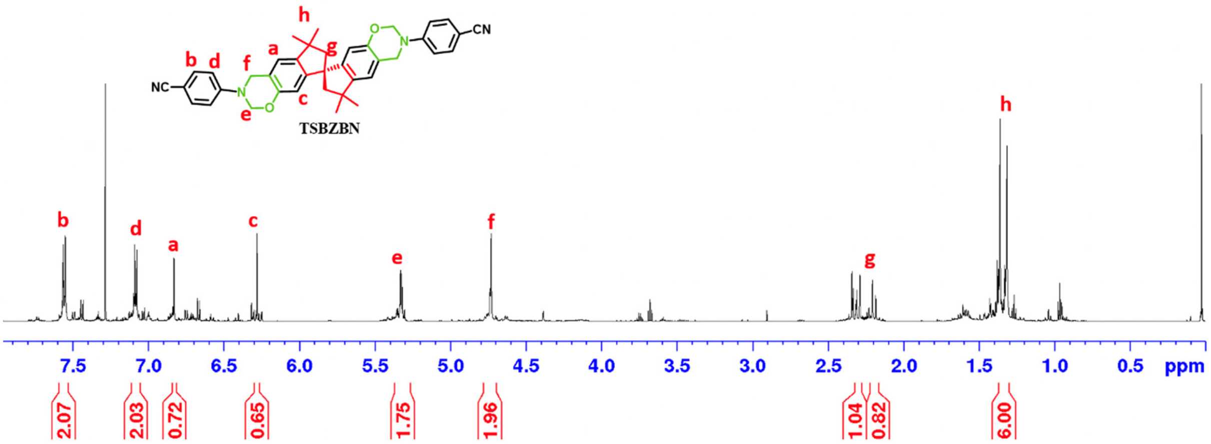

Figure S2. ^{1}H NMR spectrum of TSBZBN.

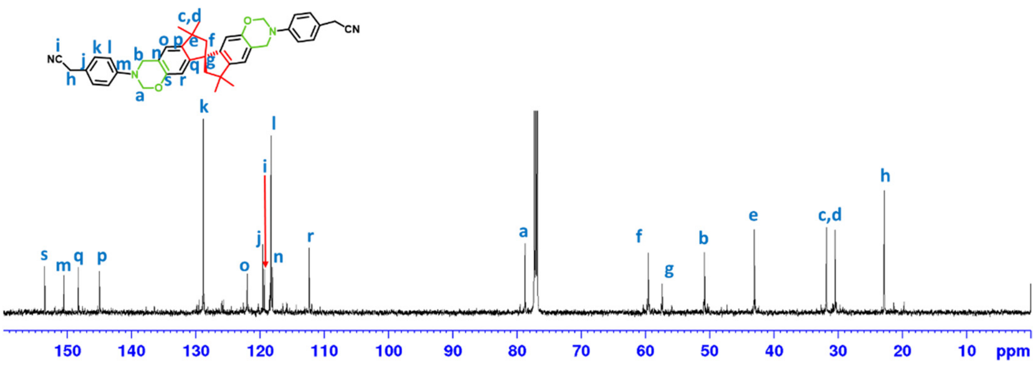

Figure S3. The ^{13}C NMR spectra for TSBZBC.

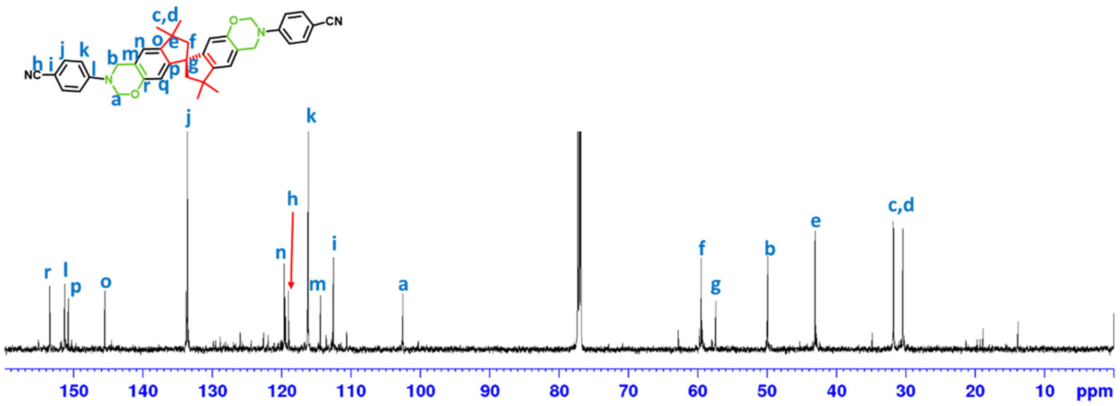

Figure S4. The ^{13}C NMR spectra for TSBZBN.

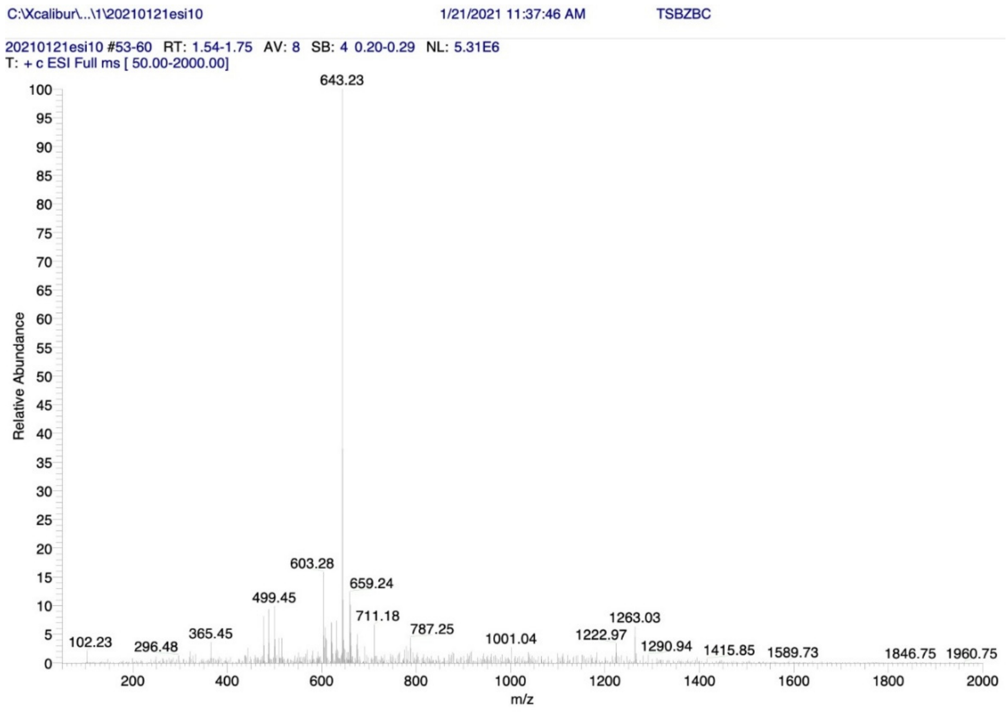

Figure S5. The ESI mass spectra of TSBZBC.

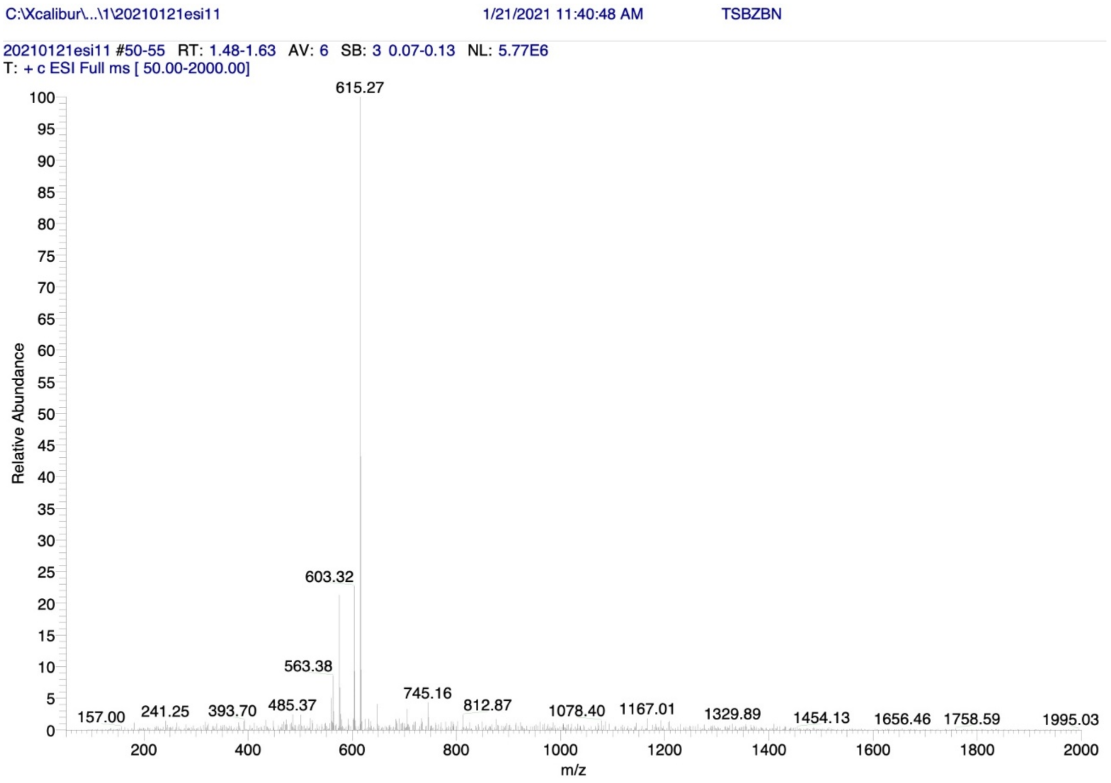

Figure S6. The ESI mass spectra of TSBZBN.

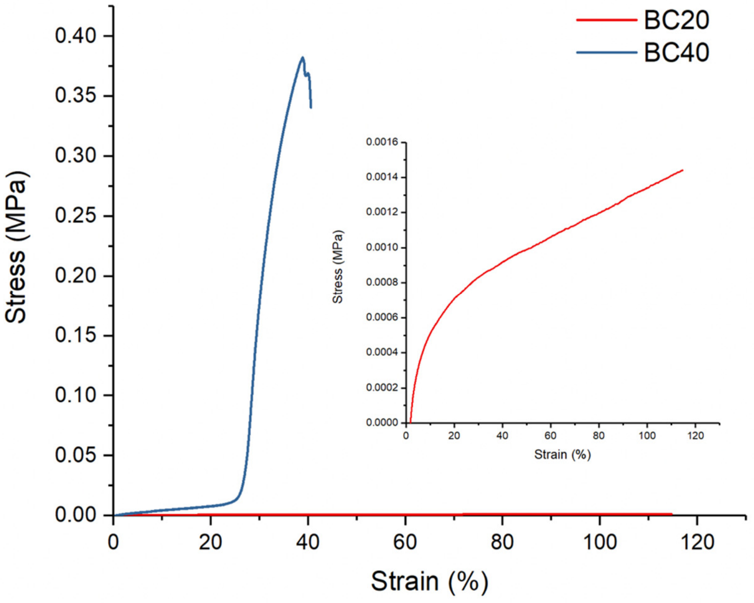

Figure S7. DMA stress−strain curves of the BC20 and BC40 sample.

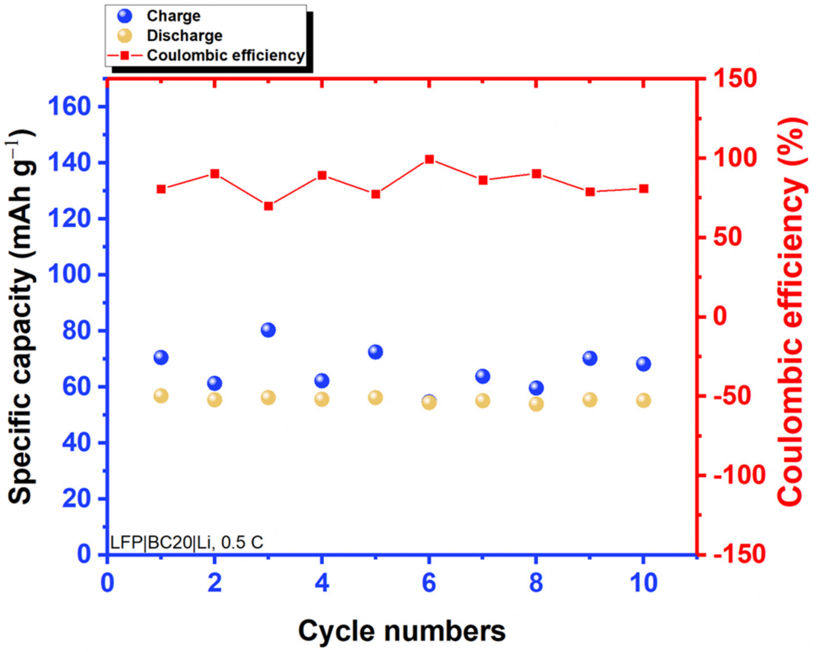

Figure S8. Cycling performance of the LFP|BC20|Li cell at 0.5 C (80 °C).

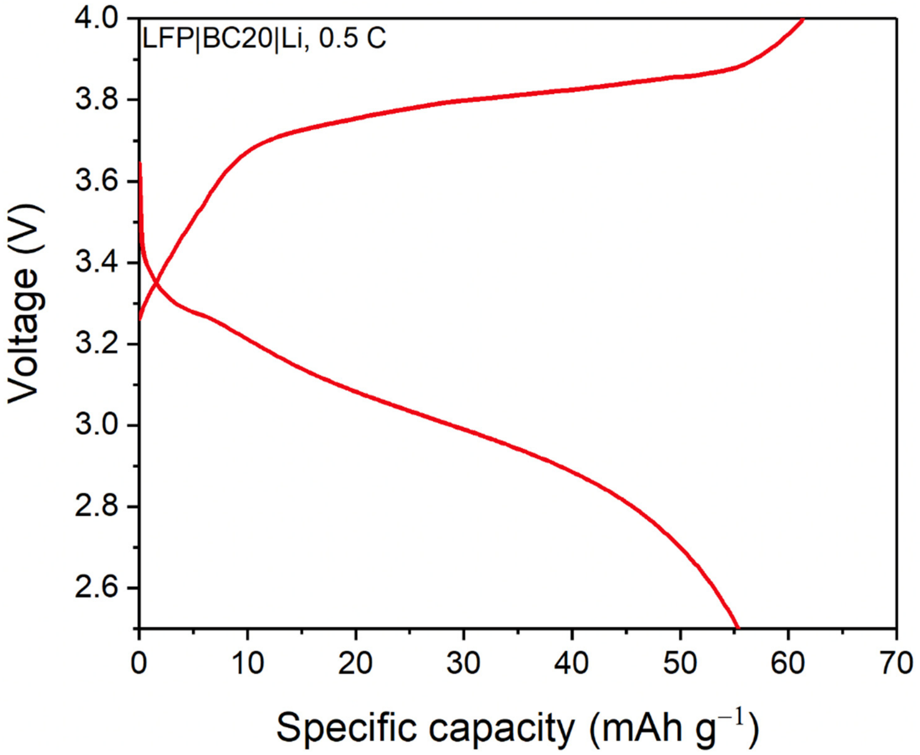

Figure S9. Charge/discharge profile of the LFP|BC20|Li cell at 80 °C.

Table S1 The ionic conductivities result of the BC10, BC20, BC30, and BC40 samples at various temperatures.

Ionic Conductivities (S cm^{-1}) \ Temperature (ºC) / SPE	RT	40	60	80
BC10	2.27×10^{-5}	1.33×10^{-4}	4.48×10^{-4}	9.32×10^{-4}
BC20	7.04×10^{-6}	3.50×10^{-5}	1.42×10^{-4}	3.24×10^{-4}
BC30	3.63×10^{-6}	2.20×10^{-5}	7.50×10^{-5}	2.44×10^{-4}
BC40	3.03×10^{-6}	1.23×10^{-5}	5.63×10^{-5}	2.41×10^{-4}

Table S2. Comparison of SPEs in this work and those reported in literature.

**Electrolyte**	**Transference number**	**Conductivity (S cm^{-1})**	**Lithium plating/stripping tests (current density [mA cm^{-2}]/cycle time [h])**	**Discharge capacity (mAh g^{-1})**	**Operating temp. (°C)**	**Ref**
**BC20**	0.187	3.23×10^{-4}	0.1/2700	158.4 (0.1 C)	80	This work
**BN20**	0.143	2.63×10^{-4}	0.1/900	153.4 (0.1 C)	80	This work
**PT20**	0.17	3.53×10^{-4}	0.1/200	165.6 (0.1 C)	80	[29]
**PEO/LiTFSI**	-	5.58×10^{-4}	0.1/144		80	[29]
**T1-00(PEO/ LiTFSI)**	0.32	5×10^{-4}	0.1/130	158 (0.05 C)	80	[65]
**T1-20**	0.43	5.61×10^{-4}	0.1/450	156 (0.05 C)	80	[65]
**PEO/LiTFSI**	0.18	1×10^{-3}	-	-	80	[68]
**PEO/LiFSI**	0.14	$>1 \times 10^{-3}$	-	146 (0.2 C)	80	[68]

**3PEG-SSH**	0.32	1.78×10^{-4} (80 °C)	-	135 (0.1 C)	60	[69]
**PEO-PEGDA-DVB-LiTFSI**	0.21	1.4×10^{-4}	-	138 (0.1 C)	70	[70]
**PEO_8–LiPCSI**	0.84	7.33×10^{-5}	0.01/1000	141 (0.1 C)	60	[71]
**PTSPE**	0.36	2.3×10^{-4}	0.1/500	~165 (0.1 C)	60	[72]
